# Leukemia Inhibitory Factor: An Important Cytokine in Pathologies and Cancer

**DOI:** 10.3390/biom12020217

**Published:** 2022-01-27

**Authors:** Megan M. Jorgensen, Pilar de la Puente

**Affiliations:** 1 Cancer Biology and Immunotherapies Group, Sanford Research, Sioux Falls, SD 57104, USA; Megan.Jorgensen@SanfordHealth.org; 2 MD/PhD Program, University of South Dakota Sanford School of Medicine, Sioux Falls, SD 57105, USA; 3 Department of Surgery, University of South Dakota Sanford School of Medicine, Sioux Falls, SD 57105, USA

**Keywords:** Leukemia Inhibitory Factor (LIF), cancer, LIFR, IL-6 cytokine family, cancer progression, chemoresistance

## Abstract

Leukemia Inhibitory Factor (LIF) is a member of the IL-6 cytokine family and is expressed in almost every tissue type within the body. Although LIF was named for its ability to induce differentiation of myeloid leukemia cells, studies of LIF in additional diseases and solid tumor types have shown that it has the potential to contribute to many other pathologies. Exploring the roles of LIF in normal physiology and non-cancer pathologies can give important insights into how it may be dysregulated within cancers, and the possible effects of this dysregulation. Within various cancer types, LIF expression has been linked to hallmarks of cancer, such as proliferation, metastasis, and chemoresistance, as well as overall patient survival. The mechanisms behind these effects of LIF are not well understood and can differ between different tissue types. In fact, research has shown that while LIF may promote malignancy progression in some solid tumors, it can have anti-neoplastic effects in others. This review will summarize current knowledge of how LIF expression impacts cellular function and dysfunction to help reveal new adjuvant treatment options for cancer patients, while also revealing potential adverse effects of treatments targeting LIF signaling.

## 1. Background

Leukemia Inhibitory Factor (LIF) is a member of the Interleukin 6 (IL-6) cytokine family [1]. The LIF receptor forms a heterodimer with gp130 upon LIF binding, which activates tyrosine kinase signaling pathways to cause changes in gene transcription. The structure, receptor binding, and initial downstream pathway activation of LIF have been heavily characterized [2], while its role in normal physiological functioning and various pathological conditions are still being elucidated. Studies of LIF have shown that it can have a wide variety of impacts on cellular function, and its pleiotropy is cell-type specific. Reviewing the literature on the potential effects of LIF in physiological and pathological conditions reinforces studies that have found similar effects of LIF within various cancer types. 

Cancers are the second leading cause of death globally [3] as well as in the United States [4], so researchers are constantly working for a better understanding of why cancers occur and how to better treat and cure them. Although there have been nearly one thousand papers written that examine the actions and expression of LIF within various cancers, there have been limited clinical trials that have investigated a therapeutic aimed at altering LIF signaling for improving outcomes of cancer patients. Examination of the impacts of LIF expression in various tumor types, while also considering its roles in normal tissue and other pathological states, is necessary to better understand the entirety of LIF signaling for the creation of effective LIF-based therapeutics. This review aims to link physiological and pathological roles of LIF to roles that it has been found to have in cancers, and to point out that additional research into the drivers, mechanisms, and downstream effects of LIF signaling is warranted.

## 2. LIF, an Important Cytokine

LIF, an IL-6-type cytokine, has variable structure and molecular weight (between 38–67 kDa), depending on the extent of its glycosylation [5]. LIF has also been known as Human Interleukin for DA cells, Cholinergic Differentiation Factor, Differentiation Inhibitory Activity, Hepatocyte Stimulating Factor, and Melanoma-Derived LPL Inhibitor [2]. Its final name of Leukemia Inhibitory Factor (LIF) is due to early research that discovered its ability to induce differentiation and inhibit proliferation of a myeloid leukemia cell line [6], but it has since been noted to have many additional functions. Differing isoforms of LIF mRNA include LIF-D (secreted), LIF-M (secreted and intracellular), and LIF-T (intracellular), which result from different initiating codons, and these isoforms allow LIF to exert autocrine and/or paracrine effects [7,8]. In a non-human primate cell line and a human embryonic stem cell line, intracellular LIF-D was seen to localize to the golgi apparatus or endoplasmic reticulum, which fits as these structures would prepare it for secretion. Of note, although LIF-D is considered to be the secreted form, it was also seen to be colocalized with Hoechst staining, suggesting potential nuclear localization, which is noted to be possible due to a conserved sequence with homology to the p53 nuclear localization sequence. LIF-T was seen to be in the same subcellular locations, but also diffusely located in the cytoplasm [9]. The exact ratio of expression of the different isoforms has been seen to be cell-type dependent and species-dependent, with the most abundant isoform in human cells typically being LIF-D or LIF-M [10]. These differences could account for some of the pleiotropy of LIF, and suggest that the transcripts could have distinct biological roles, although determining the specifics of these roles may be difficult due to the differences between species.

Although LIF is a member of the IL-6 cytokine family, and LIF and IL-6 can have some redundant actions [11], the functions, regulation, and effects of LIF can also be distinct from IL-6. In certain contexts, up-regulation of LIF reduces production of IL-6 [12] or upregulates the IL-6 receptor [13]. LIF represses viral gene expression of HPV-16, thereby decreasing cervical carcinoma cell proliferation. Conversely, treatment of these cells with IL-6 was seen to increase cell proliferation, while still repressing viral gene expression [14]. LIF significantly increases STAT3 activation within choriocarcinoma cells, while IL-6 has little to no effect [15]. Within T cells, IL-4 increases IL-6 expression but decreases expression of LIF [16]. Within pancreatic ductal adenocarcinoma, LIF inhibits the Hippo/YAP signaling pathway, while IL-6 is seen to have no effects on this pathway [17]. These differences highlight that tissue-specific and disease-specific modifications need to be studied in the context of LIF and not just IL-6 alone. 

**Figure 1:**
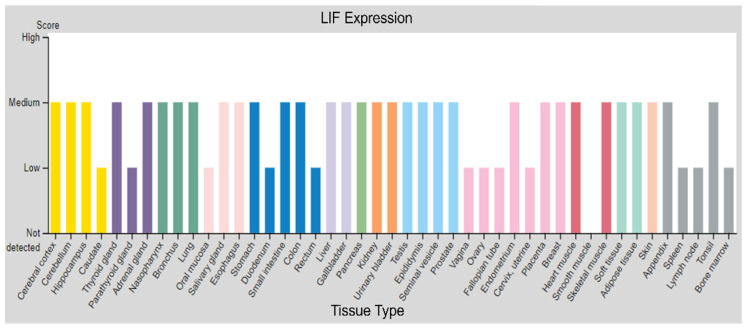
LIF expression in human tissues. LIF expression measured in a wide variety of tissue types based on Immunohistochemical staining. Figure available from Human Protein Atlas, LIF Tissue Atlas, Protein Expression Overview. v20.proteinatlas.org/ENSG00000128342-LIF/tissue (accessed on 2 May 2021).

LIF is produced by nearly every cell and healthy tissue type, as can be seen in data gathered by the Human Protein Atlas (Figure 1) [18,19]. Within cancers, LIF mRNA is expressed by epithelial carcinoma cells [20] and surrounding stromal cells (fibroblasts, monocytes, T-cells, and macrophages) [21]. Within breast tissue, LIF contributes to normal human breast epithelial cell growth [22], so it is understandable that dysregulation of LIF could contribute to breast cancer. Studies of breast cancer cell lines have shown that cancer cells internalize LIF, which stimulates growth and colony formation of cancer cells, but LIF has little effect on normal breast epithelial cells [23]. In addition to cancer cells themselves, fibroblasts within the tumor microenvironment are major producers of LIF in oral squamous cell carcinoma [24], while mesenchymal stem cells are important producers of LIF within ovarian cancer [11]. Within pancreatic cancer tumors and mouse models, both cancer and stromal cells express LIF, but only stromal cells, mainly macrophages and fibroblasts, secrete LIF [25]. 

### 2.1. LIF Regulation

**Figure 2:**
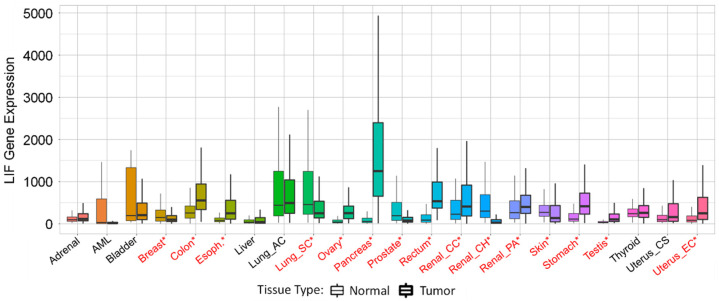
LIF expression in normal tissue and cancer. LIF expression in normal tissue compared to tumor tissue across many tissue types. Significant differences between normal and tumor tissue determined by Mann–Whitney U test are marked with red*. Figure generated from https://www.tnmplot.com/ (accessed on 3 May 2021), Copyright ©: Department of Bioinformatics, Semmelweis University 2020. Data used in figure gathered from RNAseq data from GTex, TCGA, and TARGET.

**Figure 3:**
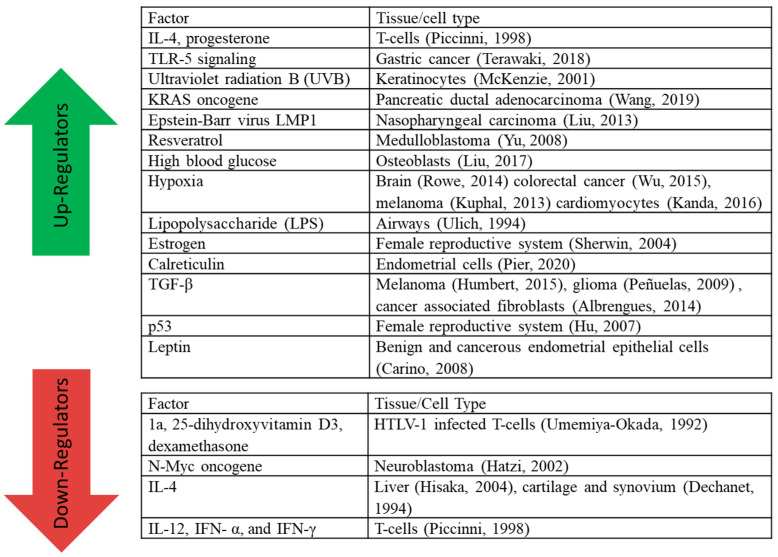
Regulators of LIF Expression. Up-Regulators and Down-Regulators of LIF expression, and the tissue types this effect is seen in.

LIF expression is often either upregulated or downregulated in cancer tissue compared to normal tissue, as can be seen in Figure 2 [26]. The level of LIF expression can have many regulators that may differ based on cell type or conditions, shown in Figure 3 [7,16,17,27,28,29,30,31,32,33,34,35,36,37,38,39,40,41,42,43,44,45,46]. Many of these up-regulators and down-regulators could contribute to the levels of LIF in tissues other than those listed, but conclusions should not be drawn until the interaction is tested in the tissue of interest. Highlighting this need for caution is IL-4, which can be a down-regulator or an up-regulator of LIF expression depending on the tissue type. These regulators exert their effects by causing LIF to be more or less likely to be transcribed, which can be accomplished through epigenetic changes, changes in transcription factor levels, and increasing LIF degradation. Within some breast cancer cell lines, DNA hypomethylation results in increased transcription of the LIF gene, and this epigenetic upregulation contributes to cancer progression. In normal breast epithelium, these DNA regions are hypermethylated [47]. Transcription of LIF can be initiated via transcription factors binding to the Smad binding element of the LIF gene promoter following TGF-β signaling [42]. For specific isoforms, the LIF-D promoter has a TATA box consensus sequence, but the LIF-M and LIF-T isoforms do not; instead, they have binding sites for Sp1 and Ets-1 [10]. The mannose-6-phosphate/insulin-like growth factor II receptor is able to bind to extracellular LIF (LIF-D or LIF-M) and internalize it, which results in rapid degradation without any signal transduction [48]; therefore, altering levels of this receptor is an alternate mechanism for controlling levels of secreted LIF.

### 2.2. The LIF Receptor

**Figure 4:**
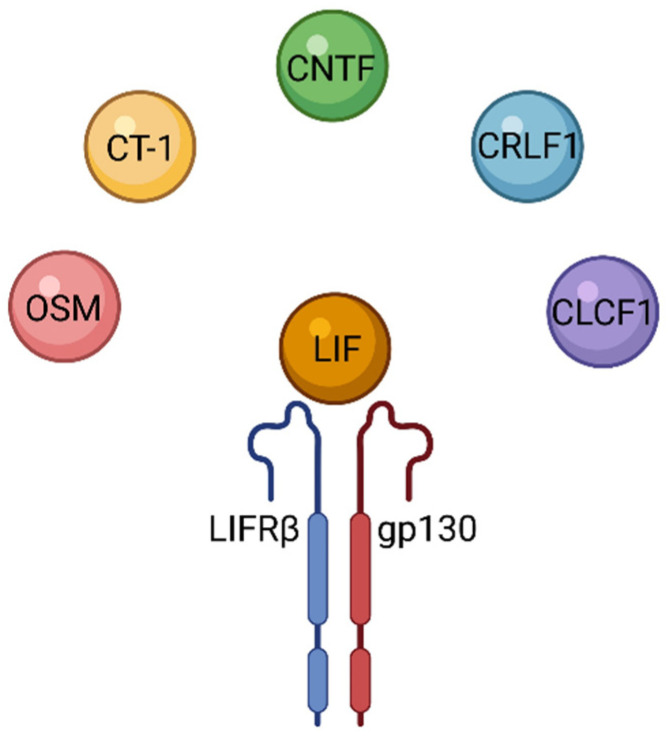
LIFR and its cytokines. The LIFR consists of LIFRβ and gp130. While LIF is the primary ligand of the LIFR, other cytokines such as OSM, CT-1, CNTF, CRLF1, and CLCF1 can also bind to and signal through LIFR. Figure created with BioRender.com.

LIF uses a receptor complex (LIFR, also known as CD118) composed of the LIF receptor β and gp130. The LIFR complex can also be used by other signaling molecules, which can be seen in Figure 4 [49]. The IL-6 family of cytokines includes IL-6, IL-11, IL-27, LIF, Oncostatin M (OSM), Ciliary Neurotropic Factor (CNTF), Cardiotrophin 1 (CT-1), Cadiotrophin-like cytokine (CLC), and additional related cytokines. These cytokines all signal through homodimers or heterodimers using the IL-6 receptor gp130 [1]. LIF and LIFR have an entropically driven interaction, and kinetic analysis of their interaction shows the on-rate to be near diffusion-limited, while the off-rate suggests a possible half-life of more than 24 h [2]. However, these studies were performed at 4 °C, so physiological values could differ. 

Just like LIF, the LIFR is expressed in a wide variety of organs and cell types. LIFR not only functions to transmit the signaling of LIF and the previously mentioned cytokines, but also has a soluble form capable of inhibiting LIF activity by preventing its binding to membrane-bound receptors, thereby inhibiting signal transduction [50,51]. Soluble LIFR may function to ensure that localized increases in LIF do not exert their effects elsewhere in the body [52,53]. Studies have shown that increased LIF will upregulate expression of LIFR [54], but that prolonged LIF stimulation can result in LIFR endocytosis and lysosomal degradation [55]. The effects of whole-body excess, deficiency, or knockout of LIF and its receptor have been studied in mice [53,56], but it is unknown how well these studies would translate to humans. LIF overexpression in mice leads to a fatal cachexic phenotype with excess bone formation, male absence of spermatogonia, and female absence of a corpus luteum, while LIF^-/-^ mice were relatively normal aside from female infertility (able to be rescued by LIF injections), hippocampal abnormalities, decreased hematopoietic stem cells, and excess olfactory neural cells [53,56]. LIFR^-/-^ mice have perinatal lethality with deficits in neurodevelopment [55,56]. Of note, congenital mutations of the LIFR gene are seen to cause Stüve-Wiedemann syndrome, which presents phenotypically with skeletal abnormalities such as bowed long bones and joint restrictions, dysautonomia, and respiratory and feeding difficulties [57]. These patients have very high neonatal mortality, with few case reports of patients surviving to childhood or adolescence [58]; therefore, conclusions regarding lifetime cancer risk and LIFR signaling are unable to be drawn from studying this genetic syndrome. However, the rare syndrome gives insights into the important roles of LIFR throughout the body and into potential risks and side effects of pharmacological interference with LIF signaling. 

**Figure 5:**
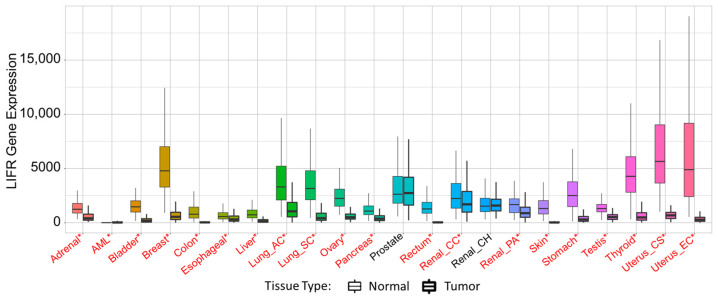
LIFR expression in normal tissue and cancer. LIFR expression in normal tissue compared to tumor tissue across many tissue types. Figure generated from https://www.tnmplot.com/ (accessed on 3 May 2021), Copyright ©: Department of Bioinformatics, Semmelweis University 2020. Significant differences by Mann–Whitney U test are marked with red *. Data from figure gathered from RNAseq data from GTex, TCGA, and TARGET.

The LIFR has also been investigated within cancers, where like LIF, its expression is often dysregulated compared to healthy tissue (Figure 5) [26]. The LIFR has been found to be a breast cancer tumor suppressor and is typically downregulated in breast cancer. In experiments with breast cancer cell lines, overexpression of membrane-bound LIFR significantly reduced metastasis through Hippo/Yap signaling [59,60]. Inhibition of the LIF/LIFR/JAK1 pathway or inhibition of BRD4, which increases LIFR transcription, increases sensitivity of breast cancer cell lines, xenografts, and allografts to histone deacetylase inhibitors [61]. LIFR has also been identified as a suppressor of hepatocellular carcinoma [62], where its expression is controlled by changes in DNA methylation [63]. Conversely, within melanoma, LIFR overexpression is associated with a poor prognosis [64]. 

Recent investigations into long non-coding RNAs have found that LIFR antisense RNA 1 (LIFR-AS1) is significantly under-expressed in Luminal B breast cancer, and lower levels are associated with a poorer prognosis [65]. Studies using breast cancer cell lines and xenografts showed that LIFR-AS1 has tumor-suppressor actions through upregulation of Sufu, a negative Hedgehog signaling regulator [66]. Some studies have shown LIFR-AS1 is over-expressed in gastric cancer patient tissue relative to adjacent normal tissue from the same patient, and higher levels are correlated with larger tumor size, metastasis, stage, and lower overall survival and disease free survival [67]. Supporting studies have shown evidence for LIFR-AS1 upregulating COL1A2 in gastric cancer cell lines, with resultant pro-tumor effects [68]. However, other investigations have shown opposing effects, with data from The Cancer Genome Atlas showing decreased LIFR-AS1 in gastric cancer tissue compared to normal tissues and gastric cancer patient tissue having relatively less LIFR-AS1 compared to matched adjacent tissues; moreover, potential tumor-suppressor effects were seen in experiments with gastric cancer cell lines [69]. These studies used different cell lines and different patient samples, and highlight the potential role of genetic heterogeneity in the tumor microenvironment as well as the effects of LIFR-AS1, even within the same cancer type. LIFR-AS1 has also been seen to have tumor-suppressor actions in glioma tissue and cell lines [70], non-small cell lung cancer tissue, cell lines, a mouse model [71], and colorectal cancer cell lines [72]. Pro-tumor effects of LIFR-AS1 have been seen in osteosarcoma cell lines and xenograft mouse models [73] as well as pancreatic cancer cell lines [74]. Opposing effects have been also seen in thyroid carcinoma tissue, cell lines, and xenografts [75,76].

### 2.3. Downstream LIF Signaling Pathways

**Figure 6:**
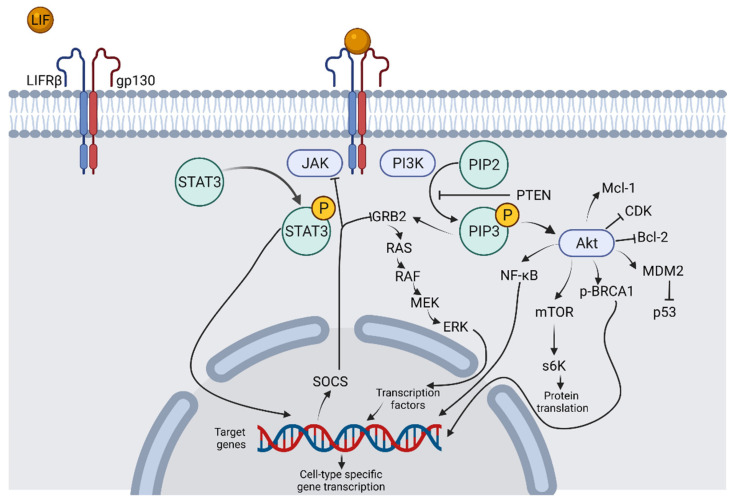
Downstream LIF Signaling Pathways. LIF binding to the LIFRβ/gp130 receptor complex activates tyrosine kinase pathways, such as JAK/STAT and PI3K, and downstream signaling molecules lead to activation of transcription factors able to alter gene expression in a cell-type specific manner. Figure created with BioRender.com.

Secreted LIF exerts its effects through binding the LIFR of nearby cells (paracrine signaling) or the same cell that secreted the LIF (autocrine signaling). Both components of the LIFR are constitutively associated with the JAK1, JAK2, and TYK2 members of the JAK tyrosine kinase family, which are activated upon LIF binding. The main kinase targeted by LIF is thought to be JAK1, which stimulates the JAK/STAT, MAPK, and PI3K pathways (Figure 6) [77,78]. The activation of these pathways often contributes to cell differentiation, survival, and self-renewal, though the effects are cell-type specific, and the balance of the individual pathway activation achieves the physiological effects. STAT3 is considered the most important downstream signal transducer of LIF/LIFR, as it is believed to mediate most of the cellular effects of LIF. An important target of STAT3 to note is SOCS3, which provides negative feedback to inhibit the JAK/STAT and MAPK signaling pathways [2]. SOCS1 is also able to inhibit signal transduction by LIF via inhibition of the JAK/STAT pathway [79]. 

**Figure 7:**
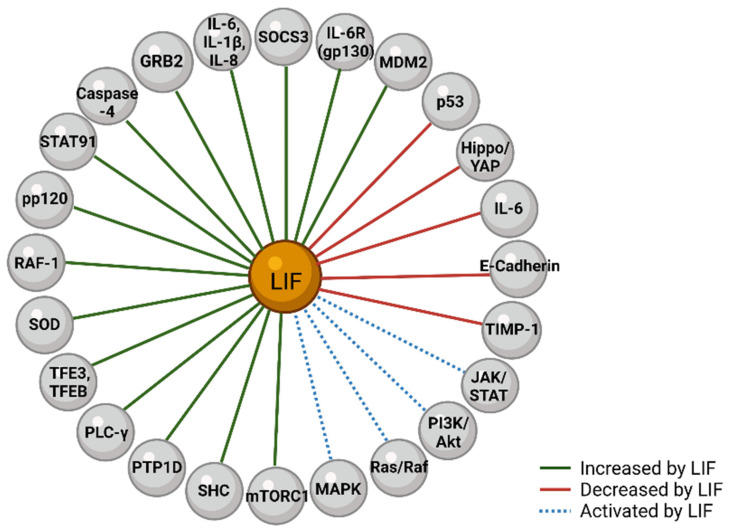
Downstream Actions of LIF. Within cancers, LIF can be seen to activate pathways such as JAK/STAT, PI3K/Akt, Ras/Raf, and MAPK. LIF expression has been linked to downregulation of p53, Hippo/YAP, IL-6, E-Cadherin, and TIMP-1. LIF expression has been seen to upregulate MDM2, IL-6R, SOCS3, IL-6, IL-1β, IL-8, GRB2, Caspase-4, STAT91, pp120, RAF-1, SOD, TFE3, TFEB, PLC-γ, PTP1D, SHC, and mTORC1. Figure created with BioRender.com.

Although the aforementioned tyrosine kinase pathways are believed to constitute most of the actions of LIF, additional pathways of LIF and their resultant effects have been noted to be of importance. Within hypoxic tissue, LIF upregulates superoxide dismutase 3 to reduce cell death [80]. Studies of nasopharyngeal carcinoma have shown that increased cytoplasmic LIF levels are associated with decreased total and phosphorylated YAP [81]. A cell-type-specific pathway that contributes to renal cell carcinoma is LIF-induced TFE3 and TFEB expression, which in turn activate E-cadherin in fibroblasts but downregulate E-cadherin in cancer cells [82]. In colorectal cancer, LIF downregulates and promotes degradation of p53 via STAT3/ID1/MDM2 [83]; conversely, p53 has been seen to be a positive regulator of LIF in medulloblastoma cells [84] and a negative regulator of LIF in leiomyoma cells [85]. Activation of the AKT/mTOR pathway by LIF signaling downstream of the PI3K pathway has been investigated as a major promoter of tumorigenesis and metastasis in breast cancer cell lines and xenograft mouse models [86,87]. Both mTOR1 and mTOR2 are well known mediators of tumorigenesis, with multiple FDA approved mTOR inhibitors currently in use as cancer treatments [88]. Inhibitors of mTOR activity have been seen to decrease LIF expression, suggesting the potential for a positive feedback loop in which increases in mTOR induce LIF expression [89]. However, inhibitors of mTOR have also been seen to increase LIF expression, suggesting potential compensatory mechanisms and highlighting LIF as a possible adjuvant treatment target to increase efficacy of mTOR inhibitors [90]. Some of the additional proteins upregulated, downregulated, or activated by LIF across certain cancer types are shown in Figure 7 [15,32,59,78,91,92]. 

The roles and mechanisms of intracellular LIF signaling (LIF-M and LIF-T) are much less understood. Within a human embryonic stem cell line, intracellular LIF was seen to be able to induce cellular apoptosis able to be inhibited by CrmA but not Bcl2, therefore, likely exerting its effects by interacting with caspase-activating pathways [9]. Fusion of intracellular LIF to an N-terminal signaling sequence is able to significantly increase levels of bioactive extracellular LIF [10]. It is also hypothesized that in the case of cell death and cell lysis, intracellular LIF would be released and would then be able to act on nearby cells, as it still has a receptor binding domain [8].

## 3. The Roles of LIF in Pathologies and Cancer

### 3.1. General Pro-Tumor Effects

Many general pro-tumor effects of LIF have been noted. During mouse and drosophila embryogenesis, dysregulated LIF signaling can result in teratocarcinoma formation [93]. LIF is an autocrine growth factor for germ cell tumor cells [8] and mediates self-renewal of glioma cells and mouse models [43]. Within breast cancer tissue, LIF expression has been seen to increase with tumor stage [94], and LIF expression in breast cancer tissue was found to correlate with relapse-free survival, suggesting high LIF expression could be a poor prognostic marker [86]. Within osteosarcomas, LIF overexpression significantly correlates with advanced stage, larger tumor size, and decreased survival [95]. High LIF expression in tumor tissue has been associated with poorer overall survival in many cancer types, such as chordomas [96], oral squamous cell carcinoma [97], nasopharyngeal carcinoma [81], pancreatic adenocarcinoma [98], cervical cancer [19,99], and renal cancer [19]. Additional cancers in which LIF has been implicated as having a role in inducing pathogenesis include Langerhans cell histiocytosis, where LIF may contribute to thrombocytosis and osteoclast activity [100]; nasopharyngeal carcinoma, where LIF is linked to tumor progression and chemoresistance [101]; breast cancer, where LIF is linked to progression, metastasis, and prognosis [86]; and skin cancers, where LIF is linked to hyperplasia and UV radiation response [102].

### 3.2. Anti-Tumor Effects

In contrast to the pro-tumor activities of LIF that have been outlined above, further proving the complexities and context-dependent effects of LIF are the anti-tumor actions that LIF has been seen to have. Within gastric cancer cells and mouse models, LIF has been seen to be downregulated, and the addition of recombinant LIF or genetic LIF upregulation inhibits proliferation and tumor progression by downregulating cyclin D1, thereby inducing G1 phase arrest [103]. LIF inhibits the proliferation of cervical carcinoma cells through downregulation of HPV-16 oncogene expression [14]. However, high levels of LIF in cervical cancer tissue have also been associated with decreased patient survival [99]. LIF is able to suppress medullary thyroid carcinoma cell line xenografts [104], likely through induction of growth arrest and cell differentiation by LIF [105]. Studies of choriocarcinoma cells have shown that LIF expression reduces cell proliferation by downregulation of micro-RNA miR-141 and upregulation of miR-21 and miR-93 [106].

### 3.3. Treatment Resistance

Experiments with chordoma cell lines have shown that LIF can induce chemoresistance by increasing expression of the ABCG2 drug transporter [96]. LIF was also found to be chemoprotective of cholangiocarcinoma cells through activating the PI3K/AKT pathway, which then upregulates Mcl-1 [21]. Within endometrial cancer cells, LIF enhances chemoresistance through increasing activity of STAT3, Bcl-2, and Bcl-xl [107]. The possibility of targeting LIF as a method of combatting chemoresistance has been studied extensively in pancreatic cancer, where genetic and pharmacologic inhibition of LIF signaling has been seen to improve chemotherapy efficacy in studies of cell lines and in mouse models [17,108]. LIF signaling within breast cancer is seen to limit the effectiveness of treatment with histone deacetylase inhibitors in cell lines and patient-derived xenograft or allograft models [61]. Within prostate cancer cells, androgen deprivation therapy was shown to increase expression of the transcription factor ZBTB46, which upregulates LIF expression. LIF in turn is seen to activate signaling cascades that contribute to neuroendocrine differentiation of prostate cancer cells, and the resultant tumor is highly resistant to chemotherapeutics [109].

Cell death due to ionizing radiation largely occurs as a result of DNA double stranded breaks and ATM kinase activation of p53-mediated cell death [110]. Within nasopharyngeal carcinoma cells and xenograft mouse models, increased LIF activates mTORC1/p70S6K signaling that suppresses the normal DNA damage responses that stimulate apoptosis or DNA repair, and thereby induces radioresistance of these tumors [32]. Further experiments to determine regulators of radioresistance in nasopharyngeal carcinoma used cell lines to identify MAP2K6 as an important regulator tied to LIF signaling-induced radioresistance [111]. Studies of esophageal adenocarcinoma cell lines and ex vivo patient tumor biopsies found that LIF was significantly upregulated in terms of secretion and intracellular expression of radioresistant cells, and radiation treatment further increases LIF secretion by cancer cells [112]. 

### 3.4. Immune Evasion

LIF was named for its effects on immune cells, though its effects on the hematopoietic system are much broader than its namesake implies. LIF has important roles in hematopoiesis, such as stimulation of platelet formation [53], and pro-inflammatory effects, such as increasing neutrophil adherence to endothelial cells [113]. However, LIF is seen to have anti-inflammatory effects in rat airways after exposure to lipopolysaccharide [39]. In mouse models of organ transplantations, LIF is one of the only cytokines that exclusively represses rejection and induces immune tolerance [114]. Cancer cells have also developed ways to evade recognition and destruction by immune cells [115], and LIF may contribute to this. LIF expression has a significant positive correlation with tumor-associated macrophages in several tumor types, and neutralizing or inhibiting LIF in organotypic models and mouse models of glioblastoma was able to induce tumor infiltration of CD8+ T cells, natural killer cells, and regulatory T cells, while decreasing tumor-associated macrophages [116]. Within esophageal adenocarcinoma ex vivo biopsies, higher tumor cell LIF secretion was significantly negatively correlated with infiltration by lymphocytes [112]. In addition, LIF has also been seen to activate polymorphonuclear myeloid-derived suppressor cells (PMN-MDSCs) in mouse models of prostate cancer [117]. PMN-MDSCs are barely detectible in healthy patients, but accumulate in cancer patients, restricting the responses and activity of the immune system [118].

### 3.5. Cachexia

Cancer cachexia is defined as ongoing loss of skeletal muscle mass, with or without loss of fat mass [119], and cachexia is a known indicator of poor prognosis for cancer patients [120]. Cytokines have been investigated as potential causes of cancer cachexia for nearly three decades, and although cachexia is likely unable to ever be attributed to one single cytokine, LIF is considered a major contributing factor to causing cachexia [121,122]. Within the hypothalamic-pituitary-adrenal axis LIF is able to stimulate POMC gene expression and ACTH secretion. ACTH causes secretion of glucocorticoids such as cortisol from the adrenal cortex, which acts metabolically to stimulate gluconeogenesis and lipolysis. Thereby, LIF impacts energy metabolism and shifts it towards weight loss and cachexia phenotypes [123]. Studies of the mouse C26 model of colon cancer have established LIF and its related cytokine, OSM, as key causal cytokines of cancer-related skeletal muscle atrophy [124]. The CRISPR-Cas9 knockout of LIF within this model resulted in significantly decreased loss of body weight, muscle, and fat, further proving the significance of LIF in causing cachexia [125]. Potential mechanisms for the role of LIF in causing cachexia include STAT3 pathways leading to activation of the ubiquitin-proteasome system [126], inducing adipocyte lipolysis [127], and JAK/STAT mediated increases in leptin levels [128]. Depending on the tissue type, leptin has also been seen to regulate LIF expression, and leptin is seen to induce greater LIF expression in cancerous endometrial cells compared to benign endometrial cells [46]. Mouse models engrafted with high LIF-secreting hematopoietic cells were seen to develop a fatal cachexia phenotype, and non-human primates injected with high doses of recombinant LIF experienced rapid loss of subcutaneous adipose, which resolved when treatment was terminated [129,130]. Further investigations with mouse models of cachexia revealed that administration of recombinant LIF could induce a short-term decrease in body weight and adipose tissue, plus a transient loss of appetite, which was compensated for in these animals by reduced leptin levels. In mice that lack leptin signaling, administration of recombinant LIF induced persistent decrease in body weight and adipose tissue, as well as a persistent decrease in appetite, suggesting LIF may be able to effect weight and adiposity through both central and peripheral mechanisms [127]. 

### 3.6. Hormone Interactions

The pleiotropy of LIF is further emphasized by its many interactions with the endocrine system. These complexities are described in detail by Auernhammer and Melmed (2000) [123] but will be briefly covered here. As is mentioned in the previous section, LIF is able to impact metabolic function and energy storage through hormonal changes in the hypothalamic-pituitary-adrenal axis, but those are not its only notable interactions with hormones. Depending on the animal model used to study the process, LIF expression can be induced by estrogen, progesterone, and/or βhCG [29,131]. LIF reduces testosterone in porcine Leydig cells [132], but is able to stimulate testosterone production in immature rat Leydig cells, with no effect in adult rat Leydig cells [133]. Therefore, in studies of LIF in animal models it is important to include both male and female animals to determine any significant differences possibly due to hormonal differences. These findings may have important implications for hormone-sensitive tumors, such as breast cancer and prostate cancer. Breast cancer can have conflicting effects of LIF, with both pro- and anti-tumor effects able to be seen [123]. One study of breast cancer saw that LIF was able to significantly increase proliferation of estrogen receptor-positive cell lines, whereas estrogen receptor-negative cell lines did not have their proliferation impacted by LIF [134]. Aside from these studies, an FDA approved selective estrogen modulator has been found to also inhibit the gp130 component of LIFR [135]. 

### 3.7. Invasion, Migration, and Metastasis

In the female reproductive system, LIF expression is necessary for blastocyst invasion into the endometrium and implantation [136,137], which supports research showing the capabilities of LIF to contribute to invasiveness of cancer cells. LIF signaling in cancer associated fibroblasts and carcinoma cells has been shown to cause extracellular matrix remodeling and actomyosin contractility to form a proinvasive environment for tumor cells [44]. High intracellular LIF is correlated with lower metastasis-free and recurrence-free survival in nasopharyngeal carcinoma, in which xenograft zebrafish and mouse models reveal that LIF enhances vascular invasion, invadopodia formation, and focal adhesion kinases via suppression of YAP1 [81]. Increased LIF can also increase invasion and migration of chordoma cell lines, through decreases in E-cadherin and CK19 and increases in ZEB2 and MET [138]. While LIFR expression is similar between different breast cancer cell lines, LIF expression is highly varied and correlates with the metastatic potential of the cell lines. Addition of exogenous LIF to cell cultures increased cell migration, invasion, and proliferation through activation of the AKT/mTOR pathway, which was able to be blocked by addition of a LIF-neutralizing antibody or shRNA knock-down of LIF [86]. Choriocarcinoma cell and osteosarcoma cell line and mouse models have shown cancer cell invasiveness is potentially due to LIF-induced STAT3 activation [15,95]. In pancreatic ductal adenocarcinoma cells and mouse models, LIF signaling enhances migration of peripheral nerve schwann cells via STAT3 and AKT pathways [25]. LIF expression within oral squamous cell carcinoma tissue promoted cancer cell invasion [24], and the inhibin beta A subunit gene is a major downstream mediator of these effects [97]. In melanomas, LIF can induce osteoclastogenesis and is associated with significantly greater incidence of metastasis to bone in mouse models [139]. Another cancer that typically metastasizes to bone is rhabdomyosarcoma, mouse models of rhabdomyosarcoma identified LIF expression within the bone marrow as a chemoattractant for rhabdomyosarcoma cells, and treatment with siRNA against LIFR was able to decrease bone metastasis. LIF expression within rhabdomyosarcoma cells increased cell adhesion and stimulated F-actin bundle formation and colocalization with FAK and paxillin in filopodia [140]. Mouse models of pancreatic cancer have shown that upregulating LIFR can significantly decrease metastasis; therefore, the LIFR may play a protective role negatively regulating metastasis [141].

### 3.8. Cancer Stem Cells

LIF is seen to have an important role in embryogenesis and the development of normal bones [142,143], thymus and hematopoietic cells [144,145,146], cardiac tissues [147,148], and neural cells [149,150,151,152,153]. These roles in development highlight the ability of LIF to influence cell fate and differentiation. It has long been known that LIF can be added to culture media to maintain pluripotency of mouse embryonic stem cells [154], and that high concentrations of LIF can maintain self-renewal and pluripotency of human embryonic stem cells [155,156]. Therefore, LIF has been believed to have a potential role in promoting dedifferentiation and self-renewal of cancer stem cells (CSCs). CSCs are considered to be important drivers of progression, epithelial to mesenchymal transition, chemoresistance, radioresistance, and metastasis of tumors [157]. LIF has been found to be a promoter of CSCs in chordoma cell lines, with upregulation of NANOG, Oct4, and Klf4 [96]. In glioma cell cultures, LIF treatment doubled the amount of CD133 positive cells, and prevented cell differentiation [43]. Conversely, within gastric cancer cell lines as well as patient-derived xenograft cells and tumourspheres, LIF has been seen to inhibit tumorigenic properties of CSCs [158]. In addition, key pathways that are induced by LIF have also been implicated as leading to survival of CSCs, even in cancers where LIF itself has not been directly implicated. For example, within medulloblastoma mouse models, the PI3K pathway has been seen to regulate radioresistance and survival of CSCs [159]. 

### 3.9. Angiogenesis

Upregulation of LIF in the endometrium is known to stimulate angiogenesis [41]. Within mice, bone-marrow-derived mesenchymal stem cells transfected to overexpress human LIF have a greater angiogenic ability, with upregulation of angiogenin, IL-8, MCP-1, VEGF, and perivascular cell markers [160]. However, LIF can also inhibit retinal vascularization in transgenic mice [161]. Mouse models of myocardial and cerebral infarction show an important role of LIF in neovascularization of myocardium after the infarct [162], as well as neuroprotective actions after a stroke [80], the actions of LIF within these ischemic pathologies reiterates the induction of LIF by hypoxia. Tumor growth can lead to hypoxic “pockets” of cells, and it has been hypothesized that the hypoxic cells may secrete LIF to stimulate angiogenesis. Experiments with choriocarcinoma cells showed that increasing LIF concentrations transiently decreases VEGF gene expression [163]. Conversely, LIF signaling increases IL-8 transcription, leading to increased angiogenic activity within colorectal cancer cells [164]. 

### 3.10. LIF as a Biomarker

Interest in using LIF levels as a predictive or diagnostic clinical tool have led to various methods of testing LIF concentrations that are less invasive than tissue biopsies. LIF levels in uterine flushings are able to predict likelihood of a successful reproductive outcome in women with infertility of various causes with high sensitivity and specificity at a cutoff point of 2.31 pg/mL [165]. LIF is elevated in patient plasma in many different cancer types, and, therefore, it has potential as a non-specific cancer biomarker (although studies are needed to determine plasma levels of LIF in patients with non-cancer pathologies in addition to the healthy control subjects typically used). Nasopharyngeal carcinoma patients with higher serum levels of LIF were more likely to have local tumor recurrence, and receiver-operating characteristic (ROC) analysis showed a cutoff value of 4.96 pg/mL could differentiate between patients with complete remission and those with recurrence [32]. Serum LIF levels of esophageal adenocarcinoma patients were significantly elevated in patients who would go on to have poor pathological response to treatment [112]. Within pancreatic ductal adenocarcinoma, circulating LIF levels correlate to disease status, and to tumor responses to chemotherapeutics [108]. Additional studies of pancreatic cancer found that ROC analysis of serum LIF levels was more effective than other biomarkers (CA199 and CEA) at predicting metastasis [166]. Patients with prostate cancer had higher levels of LIF within their serum compared to serum of patients with benign prostatic hypertrophy and healthy patients, and serum LIF levels were even more elevated in cases of metastasis to bone [109]. Serum LIF levels are significantly lower in patients with myeloid leukemia than in healthy subjects, but LIF levels are significantly greater in bone marrow plasma than peripheral blood plasma regardless of myeloid leukemia status [167,168]. 

## 4. LIF Signaling and Potential Therapeutics 

Investigating ways to manipulate LIF signaling for therapeutic purposes has resulted in production of recombinant human LIF, LIF blockers, and inhibitors of the LIF receptor. Blocking LIF signaling has been studied through utilization of neutralizing antibodies [108] (NCT03490669), high affinity soluble human LIF receptors (eLIFR-Fc) [169], and small molecule inhibitors of LIFR, EC330 and EC359. The redundancy of the IL-6 type cytokines and the fact additional cytokines are able to bind to and signal through the LIFR have caused concern that blocking LIF itself would not be able to have significant enough effects; therefore, blocking the LIFR has been investigated as a potentially more effective treatment option [170,171]. Early studies of LIFR inhibitors suggest favorable safety profiles and pharmacokinetics as well as favorable decreases in tumorigenesis of breast (EC330 and EC359) and pancreatic (eLIFR-Fc, EC359) cancer cells in vitro [172,173,174]. 

Studies showing the role of LIF signaling in infertility have generated interest in targeting this cytokine as a potential non-hormonal birth control, or increasing LIF as a treatment to improve IVF outcomes [175,176]. A phase I study has been conducted for recombinant human LIF subcutaneous administration, and the pharmacokinetics have been described [177]; further study showed that subcutaneous recombinant LIF significantly decreased clinical pregnancy rate, which was unexpected [178]. An unrelated Phase I study investigated LIF as a potential therapeutic for rescue of hematopoietic cells in patients with advanced cancer before and during chemotherapy treatment. Treatment with greater than 4.0 μg/kg/day recombinant human LIF (rhLIF, emfilermin) effectively increased platelet and neutrophil count recovery after chemotherapy. Adverse effects of rhLIF included increased c-reactive protein levels, fevers, rigor, hypotension, impotence, and autonomic dysfunction, with one instance of deep vein thrombosis. Concentrations of 16.0 μg/kg/day were considered to have dose-limiting toxicities [179]. A phase II study of rhLIF was investigated for prevention of chemotherapy-induced peripheral neuropathy, but found that it was not effective. However, they found that low dose rhLIF (2 μg/kg/day) and high dose rhLIF (4 μg/kg/day) were generally well tolerated by patients, with no adverse effects on tumor progression or chemotherapy response [180].

Additional pharmacologic mechanisms have been investigated as methods of preventing LIF-mediated effects within cancers other than targeting LIF itself or the LIFR. Blocking gp130 as a method of inhibiting signaling of all the IL-6 type cytokines has been investigated as well, and tocilizumab is an anti-IL-6R monoclonal antibody that is currently FDA approved for autoimmune arthritis, giant cell arteritis, and cytokine release syndrome [181]. There has been interest in the use of tocilizumab in cancers, but it is currently only used for prevention and/or treatment of cytokine release syndrome in patients receiving chimeric antigen receptor T (CAR T) therapy [182], although it is currently undergoing investigation in clinical trials of multiple cancer types [183]. The Src/Bcr-Abl tyrosine kinase inhibitor Saracatinib (AZD-0530) has been investigated within nasopharyngeal carcinoma as a treatment for preventing LIF-mediated increased invasiveness [81]. Raddeanin A is an attenuator of STAT3 signaling that has been investigated in many cancers as a potential drug for reversal of chemotherapy resistance [184,185]. Many additional agents that act by directly or indirectly reducing STAT3 signaling have been and are currently being investigated, some of which are FDA approved [186,187,188]. 

When considering the potential of any signaling modulators as a therapeutic, it is essential to be aware of potential adverse effects of treatment and potential toxicities. Results from the previously mentioned clinical trials of recombinant human LIF administration as a therapeutic have shown us the adverse effects of increasing LIF signaling, but there have been no completed trials to inform us of potential effectiveness or adverse effects of specifically inhibiting LIF signaling in humans. In this regard, awareness of the physiological roles of LIF is essential. Studies of LIF have shown important roles of LIF in hematopoiesis, specifically platelet formation [179], osteogenesis [142], within the central nervous system [189], and within the female reproductive system [190]. These roles are solidified by examination of case studies of the previously mentioned Stüve-Wiedemann syndrome, an autosomal recessive disorder caused by loss of function mutations of the LIFR [191]. This syndrome is characterized by bone dysplasia and osteoporosis, episodic dysautonomia, and frequent respiratory infections, often leading to death in the neonatal period or infancy [57]. Expected side effects during treatment due to systemic administration of a drug that inhibited LIF signaling could include: decreased hematopoiesis possibly leading to anemia or clotting deficiencies, osteoporosis, female infertility, and additional potential adverse effects that would need to be weighed against potential benefits of treatment. 

## 5. Conclusions: Final Remarks on LIF

LIF has long been known to be a cytokine that is extremely pleiotropic, which has made it difficult to draw conclusions about its functions. The role of LIF seems to differ greatly by the tissue that it is expressed within. As cancer is a leading cause of death, investigating potential roles of LIF to determine whether manipulation of LIF signaling could reveal novel treatment options has become increasingly important. Clinical trials that used recombinant human LIF as a treatment had relatively tolerable adverse effect profiles, but these studies did not always see the expected effects that were hypothesized based on prior studies of LIF [178,180]. Fully understanding the effects of LIF signaling within cancers and within the body as a whole offers potentials for improved cancer screening tests, cancer monitoring, and cancer treatment, as well as avenues for treatment of various other pathological processes (Figure 6). The various roles of LIF within various cancers highlight its potential as a target for adjuvant treatments, but its broad physiological roles must also be kept in mind, as disrupting LIF signaling could potentially cause a multitude of adverse effects. 

Studies and experiments that have tested effects of decreased LIF signaling by blocking, knockdown, or knockout of LIFR have limitations in that these mechanisms would also cause decreased signal transduction of the other cytokines that use LIFR, thus conclusions cannot be drawn about LIF signaling alone. In addition, studies using in vitro laboratory models of LIF signaling within cancer or other disease states may not accurately reflect what occurs within the highly complex, three-dimensional environment of the human body. Studies that use LIF knockouts help us gain insight on its function, but this approach is not yet translatable to treating diseases in humans, and similar results may not be able to be seen using other methods of decreasing LIF signaling. Research that studies effects of increased LIF expression through addition of recombinant LIF likewise have questionable translatability, as this method may not result in physiologically or pathologically relevant levels of LIF. In addition, animal models may have subtle differences in their signaling pathways that do not allow accurate conclusions to be drawn about these pathways in humans. Therefore, there is a need for additional studies to investigate LIF signaling with a focus on ensuring translatability of the results. 

**Figure 8:**
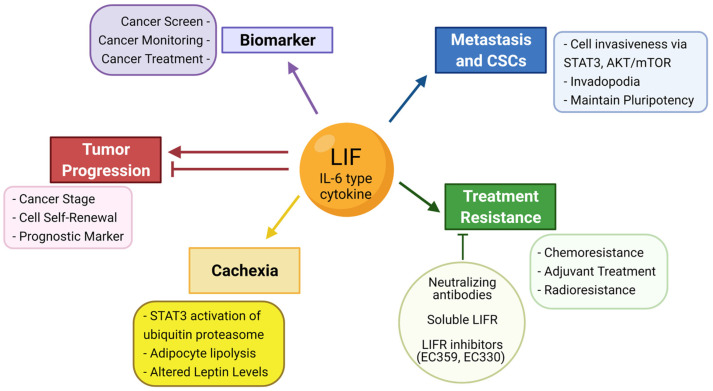
LIF in Cancers Summary. LIF, an IL-6 type cytokine, has potential as a cancer biomarker, and has roles in tumor progression, metastasis and cancer stem cells, treatment resistance, and cachexia. Figure created with BioRender.com.

Biomarker studies are limited by the relatively low number of subjects and lack of variety in the pathological conditions tested, as well as difficulty in accurately comparing measured results from tests made by different companies that may differ in their accuracy and limits of detection. The number of studies that conclude LIF is an effective biomarker for different pathologies suggests that LIF levels would not have high specificity for an individual disease, but could be useful in conjunction with more specific biomarkers in order to increase sensitivity of screening tests (Figure 8).

LIF has been or is being extensively studied within breast [192], hematopoietic [193], and pancreatic [178] cancers. While there are a multitude of studies that highlight the role of LIF in gynecological and reproductive physiology, there is a comparative lack of studies of potential roles of LIF in gynecological cancers. Roles of LIF signaling in hematopoietic cancers was studied extensively when LIF was first identified, but has not been studied in recent years and may be worth revisiting with current research techniques. Studies have shown that different cell lines of the same cancer type can have vastly different baseline levels of LIF expression [86], so use of multiple cell lines is important for ensuring translatability, as patients also are likely to have highly heterogenous tumors, and medicine is becoming more personalized. Measuring and reporting cell line LIF expression can also improve comparisons between study results and could help explain some of the conflicting results that have been seen within studies of the same cancer types that use different cell lines. Few studies in the past two decades specify the specific isoform(s) of LIF that they are studying, and narrowing down individual roles of these isoforms could provide key insights that help researchers better understand LIF’s perplexities. Mouse models have been primarily used for studying the roles of LIF within cancers, and there is a lack of studies using nonhuman primate models to further investigate these findings and provide additional evidence for potential clinical trials. A more thorough understanding of this cytokine and how it exerts its effects within cancers could reveal new treatment options for some of the most aggressive cancers and potentially improve patient outcomes and quality of life by increasing accuracy of screening tests, decreasing cancer cachexia, tapering tumor progression and spread, and reducing tumor resistance to therapeutics (Figure 8). 

## Data Availability

The data represented in Figure 1 are available from the Human Protein Atlas, v20.proteinatlas.org/ENSG00000128342-LIF/tissue. Figure 2 used RNAseq data from the GTex, TCGA, and TARGET repositories, which were accessed and analyzed through https://www.tnmplot.com/. Figure 5 used RNAseq data from the GTex, TCGA, and TARGET repositories, which were accessed and analyzed through https://www.tnmplot.com/.
